# The Protective Effect of Exercise in Neurodegenerative Diseases: The Potential Role of Extracellular Vesicles

**DOI:** 10.3390/cells9102182

**Published:** 2020-09-28

**Authors:** Oliver K Fuller, Martin Whitham, Suresh Mathivanan, Mark A Febbraio

**Affiliations:** 1 Monash Institute of Pharmaceutical Sciences, Monash University, Melbourne, VIC 3052, Australia; Oliver.Fuller@monash.edu; 2 College of Life and Environmental Sciences, University of Birmingham, Edgbaston B15 2TT, UK; M.Whitham@bham.ac.uk; 3 Department of Biochemistry and Genetics, La Trobe Institute for Molecular Science, La Trobe University, Melbourne, VIC 3083, Australia; S.Mathivanan@latrobe.edu.au

**Keywords:** extracellular vesicles, exosomes, exercise, physical activity, neurodegenerative diseases, Alzheimer’s disease

## Abstract

Physical activity has systemic effects on the body, affecting almost every organ. It is important not only for general health and wellbeing, but also in the prevention of diseases. The mechanisms behind the therapeutic effects of physical activity are not completely understood; however, studies indicate these benefits are not confined to simply managing energy balance and body weight. They also include systemic factors which are released into the circulation during exercise and which appear to underlie the myriad of benefits exercise can elicit. It was shown that along with a number of classical cytokines, active tissues also engage in inter-tissue communication via extracellular vesicles (EVs), specifically exosomes and other small EVs, which are able to deliver biomolecules to cells and alter their metabolism. Thus, EVs may play a role in the acute and systemic adaptations that take place during and after physical activity, and may be therapeutically useful in the treatment of a range of diseases, including metabolic disorders such as type 2 diabetes and obesity; and the focus of this review, neurological disorders such as Alzheimer’s disease.

## 1. Introduction

**Figure 1:**
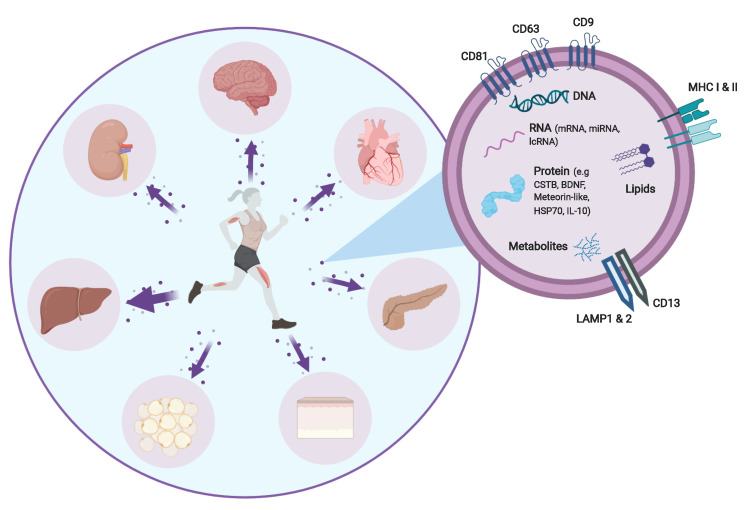
Schematic representation of inter-organ cross-talk mediated via exercise-induced extracellular vesicles (EVs) released from contracting skeletal muscle. EVs are enriched with tetraspanins, transmembrane proteins involved in transport and fusion, and contain bioactive cargo, including proteins (examples of protein cargo which might play a role in neurodegenerative diseases), DNA, RNA (mRNA, miRNA, lncRNA), lipids and metabolites.

Physical activity has systemic effects on the body, impacting most organs, including the brain. It is important, not only for general health and wellbeing, but also in the prevention of diseases. These effects are primarily due to the complex perturbation of homeostasis which takes place in all tissues as a result of the increased metabolic demands of contracting skeletal muscle. After repeated challenges, adaptations occur which are associated with improved health and wellbeing. In contrast, physical inactivity is one of the strongest risk factors contributing to chronic diseases, which make up a large portion of the disease burden world-wide, and is one of the most powerful predictors of mortality [1,2]. This has fueled interest in the potential of exercise as a treatment for disease with increasing evidence suggesting that exercise may lead to the discovery of a range of novel therapies that could target a wide range of chronic conditions, including neurodegenerative disorders [3]. In fact, it is already known that exercise can protect against age-related cognitive decline, Alzheimer’s disease (AD) and vascular dementia [4], with prospective studies indicating that higher levels of physical activity are associated with reduced risk of developing these conditions [5]. The mechanisms behind the therapeutic effects of physical activity within the brain are not well understood, however; studies indicate that these benefits are not confined to simply managing energy balance and body weight [6,7,8], but rather exercise seems to induce neuronal and vascular plasticity and possibly boost brain function through structural and neurochemical changes in the hippocampus and related medial lobe circuitry [9]. How this communication occurs is currently unknown, but recently the importance of systemic factors released into the circulation during exercise began to be fully appreciated as a means behind the myriad of benefits exercise is able to elicit around the body, including in the brain. Recently, it was shown that along with a number of classical cytokines, extracellular vesicles (EVs), specifically exosomes and other small EVs, are released into the circulation during exercise as a potential means for inter-tissue communication [10,11]. In addition, EVs are also proposed to protect the cytokines by carrying them either in the lumen or through association with the membrane to facilitate inter-tissue communication [12,13]. These EVs contain a vast array of signaling proteins and other molecules which can target specific organs, including the brain (see Figure 1). Therefore, EVs released during exercise may be one mechanism by which regular physical activity can slow or prevent the progression of neurodegenerative diseases such as AD [14,15], the focus of this review.

## 2. Alzheimer’s Disease (AD)

AD is a progressive neurodegenerative disease that affects memory and cognition. It is the most common cause of dementia world-wide, accounting for between 60% and 80% of cases [16], and is expected to increase further as the population ages. Age is the greatest risk factor for developing AD [17], and as life expectancy continues to increase due to advances in medical research and increasing awareness about the risk factors contributing to chronic diseases, the global burden of AD is expected to increase significantly. The number of people aged 60 years and over is expected to grow by 56% from 901 million to more than 1.4 billion by 2030, greatly increasing the incidence of dementia [18]. While the disease was characterised over 100 years ago, and significant progress in understanding the biology has been made, largely focused on the roles of amyloid plaques and tau pathology, there is still no effective treatment for AD.

Amyloid β (Aβ) peptide forms the major component of amyloid plaques and is derived from proteolytic cleavage of a larger precursor protein termed amyloid precursor protein (APP). APP is a type 1 membrane glycoprotein that has roles in a range of biological processes, including neuronal development, signaling and intracellular transport. It is expressed in many tissues, most predominantly in the synapses of neurons, and consists of a single membrane-spanning domain, a large extracellular glycosylated N-terminus and a shorter cytoplasmic C-terminus. It was proposed that the cleavage products of APP, specifically Aβ, caused neuronal dysfunction and thus played a role in AD [19,20,21]. Aβ peptide exists within the brain in a number of forms, the predominant form being A$\beta_{1-40}$, with A$\beta_{1-42}$ being the other common species [22,23]. It is A$\beta_{1-42}$ which makes up the amyloid plaques seen in the brains of patients with AD, however—as A$\beta_{1-42}$ has increased susceptibility to aggregation, and increased concentration within the extracellular space compared to A$\beta_{1-40}$ [24,25]. The accumulation of Aβ and its oligomers in the extracellular space was shown to cause damage through redox-active biochemical processes [26,27], induce apoptosis [28], excitotoxicity [29], inhibit LTP [30,31,32] and interfere with memory and cognition [23,33,34].

The second major hallmark of AD is the presence of neurofibrillary tangles (NFTs) composed of tau, a microtubule-associated protein which stabilizes microtubules by promoting tubulin assembly [35]. The discovery that NFTs were composed of tau increased focus on the role it played in AD. Several studies showed that the tau proteins in paired helical filaments (PHFs), the components of NFTs, in patients with AD, were abnormally phosphorylated [36,37], and that this reduced tau’s ability to bind to microtubules [38]. Aberrant phosphorylation of tau impairs cell signaling by disrupting its binding to a range of proteins implicated in signal transduction. The disruption in tau’s ability to bind to microtubules was shown to be due to a conformational change from a monomer to oligomer, caused by the hyperphosphorylation of tau, which promotes self-assembly into PHFs [39,40]. This leads to aggregation and the formation of NFTs [41,42,43,44]. The aggregates are first seen in the entorhinal cortex before spreading to the hippocampus, the limbic cortex and associated cortices [45,46], where they disrupt synaptic functioning. This is observed independently of Aβ pathology, with a large body of evidence showing strong correlations between NFTs, neuronal loss and cognitive decline, which is not observed with Aβ pathology [47,48]. Hence, that evidence, along with the lack of effective therapies targeting Aβ pathology, has led to a shift in focus onto alternative interventions fueled by a large number of epidemiological studies and long-term randomized controlled trials. These studies suggest that multidisciplinary lifestyle interventions, including exercise, can improve or maintain cognitive function and reduce incidents of AD in patients at risk of developing the disease [49,50,51,52]. Along with the increasing appreciation for the role of tau in AD pathology, is increasing evidence that the interaction between both Aβ and hyperphosphorylated tau tangles causes an immunological response within the brain.

Recent findings suggest neuroinflammation plays a much larger role in AD than previously thought. Rather than merely the by product activated in response to Aβ plaques and hyperphosphorylated tau tangles, neuroinflammation may contribute as much or even more to the pathogenesis of AD through the release of pro-inflammatory cytokines and chemokines, recruitment of peripheral immune cells and induction of various intracellular pathways [53]. Dysfunction in the blood–brain barrier (BBB) has been implicated in a range of neurological disorders, including AD [54]. The BBB is composed of capillary endothelial cells, astrocytes, pericytes, neurons and tight junctions [55]. It restricts exchange of soluble factors and cells between neural tissue and blood, ensuring the brain remains protected from harmful substances [56], while allowing certain substances such as oxygen, carbon dioxide and some soluble fats to pass through. The permeability of the BBB changes under different conditions, with increased permeability observed in patients with cerebral oedema, brain tumors and inflammation [57]. Increasing evidence suggests BBB disruption plays a role in the pathology of AD, with leakage and decreased clearance having been shown to lead to increased levels of Aβ [58]. A major cause of BBB dysfunction is inflammation, caused by inflammatory cytokines. In AD, Aβ oligomers and fibrils are able to bind to microglia via receptors (CD36, TLR4/6) resulting in activation of the microglia which start to produce inflammatory cytokines, including tumor necrosis factor-alpha (TNF-α), interleukin (IL)-1, IL-6, IL-12 and IL-18 [59]. These cytokines have been shown to play roles in inflammation and BBB dysfunction, particularly TNF-α and interleukin (IL)-1, with both having been shown to reduce BBB integrity by loosening tight junctions [60]. While sustained exposure to many pro-inflammatory cytokines impairs both degradation of Aβ fibrils via microglia and degradation of Aβ oligomers via protease degradation and paravenous clearance, recently, evidence has suggested some pro-inflammatory signaling pathways may provide benefits in a mouse model of AD [61]. Therapies or interventions such as exercise which promote activation of these beneficial pro-inflammatory pathways may reduce AD pathology by improving degradation and clearance of Aβ, thereby reducing neuronal loss and so preventing cognitive impairment.

Epidemiological and clinical studies have indicated that the risk of AD is increased in patients with type 2 diabetes mellitus (T2DM), with patients estimated to be up to two fold more likely to develop AD compared with healthy individuals [62,63]. This risk seems to be driven by peripheral hyperinsulinemia, insulin resistance and microglial inflammation [64]. Insulin has a wide range of functions within the CNS, including regulation of neuronal growth and survival, synaptic remodeling, differentiation and migration processes and modulation of neurotransmitter release. Insulin can be produced in the CNS and enter from the periphery, crossing the BBB via an active transport mechanism. In patients with hyperinsulinemia, insulin levels within the CNS are lower due to both reduced transport of insulin across the BBB and lower CNS insulin production. This correlates with lower expression of insulin receptors and insulin receptor substrates [65]. The level of insulin within the CNS is inversely correlated to the severity of AD and neurodegeneration, suggesting that impairment within the insulin signaling pathway plays a role in the pathology of AD.

## 3. Exercise and Tissue Cross Talk

Exercise has been implicated as a lifestyle intervention that can reduce the risk of a range of disorders, including cardiovascular disease, diabetes, certain types of cancer, osteoporosis and neurological disorders, including dementia [66,67]. While the epidemiological evidence is clear, the molecular mechanisms underpinning the benefits exercise provides are poorly understood, with various mechanisms being attributed to the positive effects. These include decreased inflammation, increased cardiovascular fitness, reduced adiposity and increased muscle mass [68]. Exercise causes a significant change in metabolic homeostasis, modulated by a range of factors derived from muscle and via hormonal signaling. These include the release of cytokines known as myokines; nitric oxide (NO); ATP from myofibers; hormones including glucagon released from the pancreas; catecholamines; and growth hormone released from the pituitary gland. These changes ensure adequate energy supply to contracting myofibers by releasing glucose from the liver; releasing ketone bodies; and increasing the rate of lipolysis in adipose tissue [69]. The change in metabolic homeostasis and the subsequent processes which regulate energy supply also release a number of metabolites which can act as energy substrates within the CNS in place of glucose, providing fuel to neuronal cells [70]. They also act as signaling molecules, influencing neuronal activity, formation of memories, calcium signaling, angiogenesis and myelination [71,72]. Exercise induces a number of changes within skeletal muscle, including depletion of ATP and O${}_{2}$, changes in the ratio of NADH/NAD${}^{+}$, elevation of Ca${}^{2+}$ and mechanical stress (for a comprehensive review see [73]). These lead to the activation of a number of signaling pathways, including activation of the peroxisome-proliferator-activated receptor γ coactivator 1α (PGC-1α) and peroxisome-proliferator-activated receptor δ (PPARβ/δ) axis, and a number of kinases pathways (including AMP-activated protein kinase and deacetylases). The PGC-1α-PPARβ/δ axis plays a role in the regulation of muscle mitochondrial metabolism and mediates the increase in mitochondrial biogenesis that occurs during exercise adaptation [74]. It also coordinates a number of other processes, including lactate homeostasis [75], angiogenesis through the induction of vascular endothelial growth factor (VEGF) [76] and remodeling of the neuromuscular junction via neurturin [77].

### 3.1. Myokines

During exercise, skeletal muscles produce cytokines known as myokines upon contraction. The first bone fide myokine, the cytokine IL-6, was identified by Febbraio et al. and was shown to play a role in regulating the release of hepatic glucose [78]. Hundreds of myokines have since been identified with various functions, including regulation of energy supply, muscle proliferation, differentiation and regeneration. A number of myokines have also been found to play functional roles in biological processes within the brain, with the myokines fibronectin type III domain-containing protein-5 (FNDC5)/irisin and cathepsin B (CTSB) both being upregulated within the hippocampus after exercise [79].

Irisin, the secreted form of FNDC5, a membrane protein, originates from skeletal muscle and has a wide range of biological effects including the beigeing of white adipocytes; increasing energy expenditure; and modulating muscle and liver cell metabolism. Irisin also plays several roles in the brain, including neural differentiation, and neurogenesis and memory formation through the stimulation of brain-derived neurotrophic factor (BDNF) in the hippocampus [80,81,82,83,84]. Irisin is a PGC-1α-dependent myokine, which when induced by exercise causes the release of BDNF in a cell-autonomous manner. When treated with recombinant BDNF, expression of FNDC5 decreased, suggesting this process is regulated via a negative feedback loop [80]. Whether peripheral irisin could cross the blood–brain barrier (BBB) and mediate these effects within the brain or whether the effect was due to brain-derived irisin was unknown until a subsequent study in mice showed that by over-expressing FNDC5 via an adenoviral vector in the liver, circulating levels of irisin were increased and led to increased BDNF expression in the hippocampus, suggesting irisin can cross the BBB and affect expression of BDNF, providing a mechanism for exercise-induced neurogenesis [80]. A recent study suggested this increase in neurogenesis mediated via muscle derived irisin is important for regulating synaptic function and improves cognitive function in a mouse model of AD [85].

CTSB is a cysteine protease, ubiquitously expressed throughout the body, including in skeletal muscles. Exercise has been shown to increase plasma concentrations of CTSB in both human and animal studies. Both voluntary running and treadmill exercise significantly increase plasma CTSB concentrations in mice, monkeys and humans. As with irisin, whether CTSB could cross the BBB or had any biological function within the brain was initially unknown. Moon et al. showed that CTSB could cross the BBB and when applied to hippocampal progenitor cells enhanced expression of BDNF. Compared with WT mice, when CTSB was deleted, running did not enhance adult hippocampal neurogenesis or improve spatial memory function, suggesting CTSB plays a large role in mediating the beneficial effects of exercise on cognition [86]. In humans, plasma levels of CTSB have been shown to weakly correlate to fitness and memory score, but whether this leads to improvements in cognition and memory or would have any therapeutic benefit in neurodegenerative diseases such as AD has not been explored yet [86].

## 4. Exercise and AD Protection

Physical activity and increased fitness levels are associated with maintenance or improvements to brain biology and function, reducing the risk of dementia and AD; a number of large epidemiological and prospective studies have shown that exercise reduces the risks of dementia and AD by 28% and 45%, respectively (numbers complied from several studies in a systematic review [87]). The benefit derived from exercise, not only for patients with AD but brain health generally, may be driven by a number of factors. It is well established that new neurons are able to grow within the dentate gyrus of the hippocampus and it has been shown in rodents that physical activity is able to enhance neurogenesis, with exercise having been shown to more than double the production of new neurons [88,89]. The mechanisms underlying the benefit derived from exercise on neurogenesis are not well established yet but a number of mediating components have been proposed. These include neurotrophins such as BDNF and nerve growth factor (NGF) and immune cells such as microglia which may also play a role in the increase of neurogenesis induced by exercise [90]. Many of these factors, including BDNF, VEGF and insulin-like growth factor-1 (IGF-1), are exercise responsive and, interestingly, have been independently identified within EVs [91].

### 4.1. AD Pathology

While the epidemiological evidence suggests exercise is beneficial to brain health, reduces the risk of AD and has been associated with decreased cognitive decline, the effects on amyloid pathology are less clear, with only a few studies having been done in humans. Self-reported exercise is negatively correlated with amyloid plaque accumulation in the brain, and levels of Aβ and tau in cerebrospinal fluid (CSF) in presymptomatic, autosomal dominate AD mutation carriers [92]. Other studies have found no effect on cortical Aβ levels (evaluated using PET) when patients with mild to moderate AD (assessed according to the NINCDS-ADRDA Alzheimers Criteria and DSM-IV codes [93]) undertook a 16 week exercise intervention (moderate to high intensity aerobic exercise three times weekly for one hour). In this case, the lack of efficacy may be due to the short intervention period, and relatively late stage of AD progression [94]. While changes in AD pathology have been demonstrated in a number of studies, these do not always translate to improvements in cognitive function.

### 4.2. Cognitive Function

As with AD pathology, the effect of exercise on cognitive function in humans, especially those with AD, has not been extensively explored. A number of studies have shown exercise improves pattern separation, a hippocampal dependent process [95,96,97]; and in older humans, fitness levels and hippocampal volume were correlated with performance in a spatial working memory task [9]. In older participants with mild cognitive impairment, a 12 month aerobic exercise intervention led to improvements in verbal episodic memory [98]. A number of other studies did not show improvements in cognitive function, mostly due to large inter-individual variability [9,99]. In animal models, the results are more consistent, though the improvement in cognition following exercise does not always correlate with reductions in AD pathology. Both voluntary or forced exercise leads to improvements in cognition with corresponding reductions in Aβ [100,101,102,103], and in a number of cases, without a change in Aβ pathology [101,104,105]. Whether the exercise intervention is applied chronically or acutely also seems to be an important factor determining whether a benefit is derived in mice with AD. This was shown recently by Stein et al. who showed an acute bout of exercise was beneficial in littermate WT controls, improving spatial memory, but had no effect in AD transgenic mice [106]. These findings support the findings in human studies which suggest Aβ pathology is not a reliable predictor of cognition in patients with AD, and that other factors such as neurogenesis may play larger roles in modulating cognitive decline.

### 4.3. Hippocampal Neurogenesis

Assessing neurogenesis in human subjects is difficult, with most studies quantifying the number of cells displaying neuroblast markers in postmortem brains; therefore, the process of neurogenesis is most often studied in animals. Associations, however, among cortex gray matter volume, cardiovascular fitness and memory function in young adults have been observed [107]. Aerobic exercise has been shown to increase dendritic complexity and the number of dendritic spines in the hippocampus and improve spatial memory and pattern separation in mice [108]. Exercise also reverses declining neurogenesis and memory function in aged mice [109]. This is modulated by BDNF; WT mice lacking BDNF show decreased synaptic plasticity not only in the hippocampus but also in the cortex and striatum [110], and exercise increased the levels of hippocampal BDNF consistently across multiple studies [111,112]. In mouse models of AD, voluntary and forced exercise have similar effects, enhancing neurogenesis within the hippocampus [102,113,114], though in some studies this increase was less compared with WT mice [103,115]. It was thought that the release of BDNF after exercise was confined to the brain; however, a number of studies showed that exercise elevated levels of BDNF in the blood [116,117,118]. This peripheral BDNF originates from the brain, platelets and skeletal muscle, and is mostly involved in promoting muscle fiber fat oxidation and muscle development via autocrine and paracrine signaling. Whether muscle-derived BDNF is able to cross the BBB and elicit any of its beneficial effects within the brain remains controversial. Pan et al. showed BDNF was able to cross the BBB using radiolabeled BDNF, and that it utilizes a saturable transport system. However other studies have shown minimal transport of BDNF across the BBB and that the relationship between central and peripheral BDNF is highly activity-dependent [117,119]. While the exact relationship between peripheral and brain-derived BDNF remains unclear, increasing evidence suggests there may be alternative transport mechanisms, primarily EVs, which are able to transport BDNF across the BBB [120].

### 4.4. Inflammation

It is well known that exercise has anti-inflammatory properties which decrease the risk of various cardiovascular and metabolic disorders, and increasing evidence suggests these effects extend to the brain. The anti-inflammatory properties of exercise are mediated through the release of the myokine IL-6 from contracting skeletal muscle, which up-regulates a number anti-inflammatory cytokines, including IL-10, and down-regulates pro-inflammatory cytokines, including TNF-α and IL-1β [121,122]. Exercise also affects inflammation within the brain, up regulating IL-10 and activating microglia and astrocytes. These anti-inflammatory effects have been shown to correlate with cognitive decline in mice, suggesting the modulatory effects of exercise on inflammation both centrally and peripherally have a protective effect on cognitive function and therefore may offer benefit to patients with AD [123].

### 4.5. Metabolic Dysfunction, AD and Exercise

Exercise causes a significant change in metabolic homeostasis due to the energy demands of contracting skeletal muscle. Skeletal muscle plays a critical role in glycemic control as it is the main site of glucose release when stimulated by insulin. After an acute session of exercise in healthy humans, glucose metabolism is altered; whole body insulin sensitivity improves, along with increased glucose uptake in skeletal muscle, increasing glucose tolerance [124,125,126]. Along with these improvements in energy metabolism, cognitive and behavioral functions were also improved. While it is hard to separate the mechanism responsible for this as many of these studies also showed reductions in AD pathology, patients with disorders which effect metabolic functions, such as diabetes mellitus, are at increased risk of cognitive impairment and memory loss [127], suggesting metabolic dysfunction does play a role in mediating these effects. It is therefore likely that exercise confers some of its beneficial effects in patients with AD through improved metabolic function.

## 5. Extracellular Vesicles (EVs)

Extracellular vesicles (EVs) are membrane vesicles which can be secreted by all cells. Initially thought to function as a means to eliminate cellular waste, EVs play a central role in intercellular communication not only through their ability to act as signaling vehicles but also through the transfer of bioactive cargo such as proteins, RNA (mRNA, miRNA and lcRNA), DNA and metabolites. Though various subtypes of EVs, including exosomes, ectosomes and microvesicles; apoptotic bodies; large oncosomes; migrasomes; and exomeres have been reported [128,129], the mode of biogenesis is either from an endosome or the plasma membrane. In this review, we will refer to these vesicles generally as EVs, though most of the evidence in the literature points to the involvement of small EVs, especially exosomes and plasma membrane-derived microvesicles, in exercise mediated tissue/organ cross-talk [10,11,130,131]. Small EVs that are obtained in a 100,000 g pellet after ultracentrifugation may contain a mixture of exosomes and microvesicles that are small. Hence, we will focus mainly of these two EV subtypes in the following sections. Exosomes are small EVs with a size range of 30–150 nm and are released by the cells when the multivesicular bodies (MVBs) fuse with the plasma membrane [132]. Exosomes have been reported to be secreted from a wide range of cell types, including B lymphocytes, dendritic cells [133,134], cytotoxic T cells, platelets, neurons, oligodendrocytes, Schwann cells, intestinal epithelial cells, mast and cancer cells [135,136,137,138]. Microvesicles, in contrast, are much larger in size, normally ranging from 50 to 1000 nm, and are formed through the outward budding and fission of the plasma membrane [139]. Initially they were shown to be involved in blood coagulation, originating from platelets in normal plasma and serum [140]; however, as with exosomes, they have been shown to also play a role in cell–cell communication in a wide range of cell types [141]. The evidence that EVs are not just waste disposals, but central to cell–cell communication, eliciting and mediating various signaling pathways between cell types, ignited an explosion of studies investigating the roles of EVs in various biological processes [142,143]. In addition, the cargoes of the EVs are reflective of the (patho)physiological states of the host cells, suggesting that EVs can be exploited as sources of potential biomarkers for various diseases and can be considered as mediators of disease progression [144,145]. Furthermore, the natural availability, the systemic presence and the ease of engineering them have led to several studies using EVs as drug delivery vehicles to treat various diseases [146,147].

### 5.1. Biogenesis of Exosomes and Microvesicles

**Figure 2:**
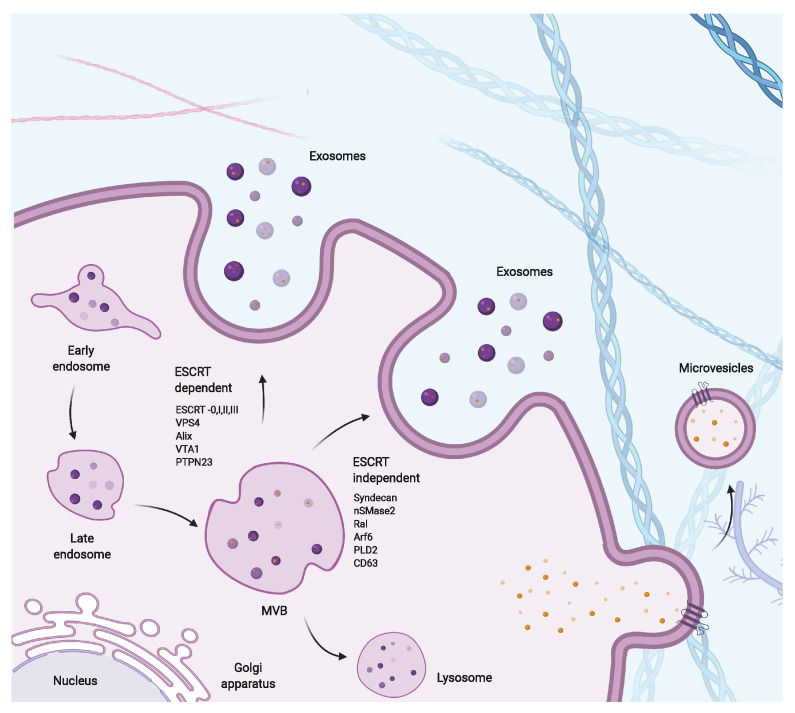
Schematic representation of pathways involved in extracellular vesicle biogenesis. Formation of EVs can occur via ESCRT-dependent and independent pathways; the related proteins involved are listed. Another mechanism of EV formation is via direct budding from the plasma membrane, forming microvesicles.

The mechanisms underlying the biogenesis of exosomes are poorly understood but relatively better understood than for other EV subtypes, including microvesicles. Both exosomes and microvesicles share similar intracellular mechanisms and cargo sorting machinery, limiting the ability to distinguish between subpopulations of EVs [148]. A schematic representation of the biogenesis of exosomes and microvesicles is shown in Figure 2. The biogenesis of microvesicles involves rearrangement of the plasma membrane and underlying cytoskeleton, which leads to budding and formation of microvesicles [141,149]. For exosomes, MVB formation is promoted by endocytosis; upon maturing into late endosomes, they recruit sorting complexes, which leads to the generation of intraluminal vesicles (ILVs) within the MVBs [150]. The sorting complexes segregate the intended cargo on the microdomains of the membranes of the MVBs, which leads to budding and fission of this microdomain into ILVs. This process is driven by endosomal sorting complexes required for transport (ESCRT) machinery, in a step-wise fashion (for a detailed description see [151]). Exosomes can also be formed in an ESCRT-independent manner with a number of studies showing depletion of the ESCRT subunits still led to the formation of exosomes. In addition to proteins, exosomes also contain DNA and RNAs, including mRNAs, and miRNAs [152,153,154]. However, the packaging of DNA inside exosomes has been often debated, with several groups claiming them to be associated in the membrane or not part of the EVs, whilst others have confirmed their detection in the lumen of EVs [152,153,154,155]. Perhaps the packaging of DNA into exosomes is cell-type-dependent and cannot be generalized to all cells. Nevertheless, the process of nucleic acid incorporation into exosomes has been shown to be regulated and selective, in addition to the passive incorporation of RNA and DNA; however, the exact contributions of these processes are currently unclear and have only been demonstrated in a few studies [156].

### 5.2. Uptake of EVs

**Figure 3:**
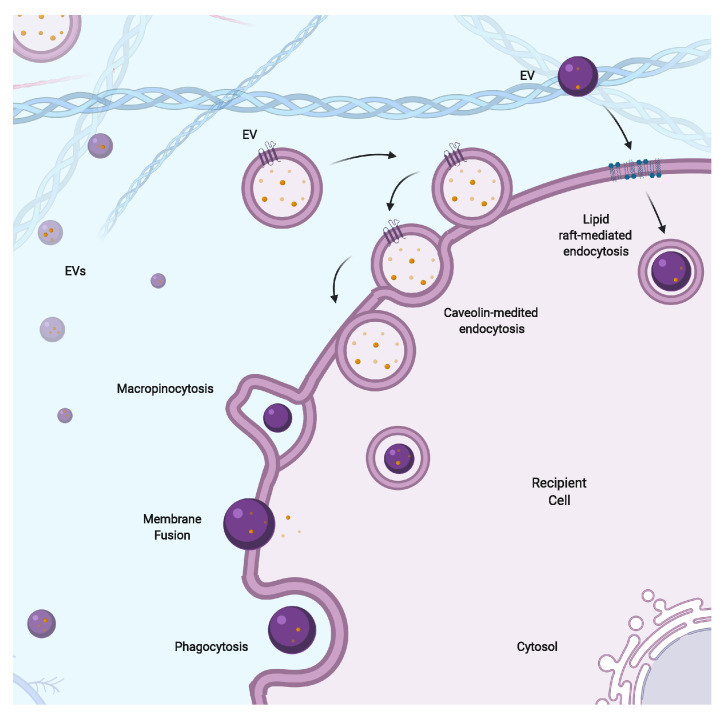
EVs are taken up by recipient cells via various mechanisms, including lipid raft-mediated endocytosis, caveolin-mediated endocytosis, macropinocytosis, membrane fusion and phagocytosis.

Once EVs are released, they elicit functional responses on the recipient cells by transferring their cargo. However, it is unclear how EVs are taken up by recipient cells. Many potential mechanisms by which EVs can be taken up by the recipient cells are shown in Figure 3. Whilst some of the uptake mechanisms have been proven with cell biology-based experiments, there is limited evidence for other pathways. For instance, receptor-mediated endocytosis seems to be the most preferred uptake route of EVs, as suggested by several studies [157,158,159,160] Regardless, the first step in this process is binding between EVs and their target cells, through specific interactions between proteins such as tetraspanins, integrins, cytokines and lipids enriched at the surfaces of the EVs and receptors such as intercellular adhesion molecules embedded on the plasma membranes of the target cells [12,161]. This has been shown to occur for a number of cell types including liver, lung, lymph node, neural and dendritic and intestinal epithelial cells [162,163,164]. Once EVs are bound to the recipient cells, they either remain at the plasma membrane or are internalized by clathrin-mediated or clathrin-independent endocytosis, such as macropinocytosis and phagocytosis, and endocytosis via caveolae and lipid rafts [146,165,166]. The content of EVs, the composition of the membrane bound proteins on their surface and the presence of specific structures on the target cell all play roles in the targeting of EVs to specific recipient cells. Once EVs are taken up by recipient cells, they follow the endocytic pathway where they can back-fuse with the endosomal membrane and release the contents to the cytoplasm [157,159] or fuse with the lysosomes [167]. Mechanisms governing these processes are poorly understood.

### 5.3. EV Signaling

EVs can elicit a functional response in recipient cells in two ways. First, EVs can activate receptors expressed on the surfaces of the recipient cells. This was first demonstrated using exosomes derived from B and dendritic cells which were able to induce an antigenic response, stimulating T cell proliferation [133,134]. The second way EVs can elicit a functional response is via the non-selective transfer of the cargo after internalization by the recipient cell. This has been demonstrated across a wide range of tissues in both healthy and pathological states, including neurodegenerative diseases such as AD. Neurons have been shown to secrete EVs which contain proteins including AMPA receptors and GPI-anchored prion protein. AMPA receptors play a role in regulating synapse function; increased secretion of AMPA receptor containing EVs after glutamatergic synaptic activation leads to decreased expression of AMPA receptors at the synapses, suggesting this is a mechanism through which plastic changes or pruning takes place [168]. Changes in EV cargo have been observed in a number of neurodegenerative diseases, including Parkinson’s disease, where EVs have been shown to carry α-synuclein between cells, transferring the disease between neurons [169]; in prion diseases where EVs have been shown to transfer prion proteins between neurons [170]; and in AD, where EVs seem to be a site of APP processing and have been shown to contain phosphorylated tau [171,172]. The exact role of EVs in AD is unclear. Whether they play a protective role, i.e., removing phosphorylated tau and processing APP, or contribute to the pathogenesis of the disease by transferring cytotoxic species between cells, is currently unclear and may depend on the stage of the disease. Along with neurodegenerative diseases, they have also been implicated in a number of other physiological and pathological processes, including inflammation, blood coagulation, tumorigenesis and more recently in the various systemic biological effects which are associated with exercise [10,173].

### 5.4. The Role of miRNAs in EV Signaling

miRNAs are regulatory small non-coding RNAs expressed by all cells; many of them are conserved across organisms with their diversity and number correlating to complexity (*C. elegans*, 437 miRNAs, mouse, 1500 and humans around 3000 miRNAs [174]). Many miRNAs are found in cells and tissues, and were considered to regulate protein abundance at the intracellular level until they were detected in the systemic circulation. Recently, however, their importance in organ to organ communication, especially via EVs, has become prominent [175]. Using liver specific DICER knockout mice (where production of miRNA in the liver is halted), a number of groups showed increased hepatic steatosis and hepatocellular carcinoma and insulin resistance compared with WT mice [176,177]. These results suggested changes in gene expression and function were not restricted to the tissue in which miRNA production was disrupted. Further studies supported this, with adipose tissue-specific DICER knockout mice showing changes in hepatic gene expression, suggesting miRNA participates in tissue cross-talk [175]. Exactly how miRNA communicated extracellularly remained unclear, until a number of studies confirmed they travel within various bodily fluids via two main routes. Valadi et al. identified miRNAs within cell line-derived EVs, which were taken up in recipient cells, releasing their contents in the process [142]. Weber et al. showed miRNAs could be transported in various bodily fluids as parts of protein complexes [178]. The finding that EVs contained miRNA was significant but not without controversy. A number of studies showed EVs could not only be used as disease biomarkers but also provide a means for intercellular and inter-tissue communication. By behaving in a similar way to hormones, miRNAs contained within EVs can alter gene expression in tissues and cells downstream of where they are produced. In support of this, recent evidence on differences in miRNA profiles between the host cells and EVs suggests that the loading of miRNAs into EVs is a selective process [179,180].

The issue of EV isolation remains a problem for determining the origin of miRNA; however, as future studies using isolation methods improve, this is expected to be less of a concern. The issue of EV isolation was highlighted in a recent study by Jeppesen et al., in which high-resolution density gradient centrifugation and direct immunoaffinity capture were used to isolate EVs from human plasma and cultured cells. Proteins involved in the biogenesis of miRNA including DROSHA and DICER were absent from EVs containing tetraspanins, including CD63, CD81 and CD9. This suggests EVs do not carry the required machinery to generate miRNA independently of cells [154]. Along with the difficulty of effectively separating miRNA contained within EVs from protein complexes carrying miRNA, in many of the studies demonstrating miRNA contained within EVs, the contribution of contamination from other sources of miRNA was unknown [181]. Hence, the detection of extracellular miRNAs may not be sufficient evidence to claim these as EV-derived miRNAs. While the exact origin of miRNA detected in EV isolates remains controversial, especially depending on the method of isolation used, increasing evidence suggests miRNA contained in exercise-induced EVs plays some role in cell–cell communication. A list of studies in which miRNA was isolated from plasma derived EVs after exercise in humans, rats and mouse models is shown in Table 1. Taking the potential targets of the miRNAs identified in exercise-induced EVs and using gene ontology, a broad range of biological pathways are significantly enriched, including response to reactive oxygen species, insulin secretion and several classes potentially relevant to neurodegenerative diseases, such as regulation of neurogenesis, specifically through axonogenesis; control of axon projection and growth; and regulation of dendrites. Furthermore, a recent examination of plasma miRNA responses to different durations of exercise observed a significant enrichment of miRNA targets involved in the neurotropin (BDNF, NGF, NT3, NT4) signaling pathway following 10 km, half and full marathon running [182]. While some of the targets of exercise-induced extracellular miRNA may play roles in neurodegenerative diseases, whether this is a mechanism through which exercise is able to illicit beneficial therapeutic effects needs to be explored. EVs carrying protein and miRNA cargo can cross the BBB both passively and via active transport mechanisms such as transcytosis and act within the brain [183,184,185]. They play a role in communication between different cell types present within the brain, including neurons, astrocytes and microglia, regulating neuronal inflammation and growth of neurons after brain injury [186], but whether peripherally derived EVs released after exercise have any therapeutic benefit in neurodegenerative diseases is currently unknown. We aim to undertake a series of experiments to discover their roles in facilitating the broad range of physiological adaptations that take place during physical activity and whether those are additional mechanisms through which exercise can protect against an array of diseases, including neurodegenerative diseases such as AD. Since skeletal muscle constitutes 30–35% body mass and has such a predominant involvement during exercise, it is conceivable that skeletal muscle is a major source of exercise EVs; that, along with the fact there is no current technique for tagging miRNA, means we are currently utilizing a skeletal muscle-specific DICER knockout mouse (MDicerKO) to investigate the role of miRNA within EVs on tissue cross-talk. MDicerKO mice and their littermate control mice undergo exercise and EVs are purified from several donor mice; the plasma is then pooled and injected into recipient mice. This strategy is allowing us to determine the effects of exercise on EV transport to other tissues and organs.

## 6. Potential Role of EVs in Mediating AD Protection

Exercise is associated with a rapid change in homeostasis, through a wide range of systemic changes occurring across the body, including changes in heart rate, blood pressure, respiration, lactate levels and circulating DNA, along with a number of long-term adaptations, such as changes in muscle metabolism, increased cardio-respiratory capacity and beneficial changes in immune function [73]. Tissue cross-talk mediated via cytokines released from tissues such as the liver, adipose tissue, brain and bone, and myokines released from skeletal muscles, has been shown to play a role in eliciting these acute and chronic physiological effects. Recently, a number of studies showed that EVs, specifically exosomes and other small EVs, are released during exercise and are able to transfer cargo to recipient tissues, providing another mechanism through which exercise can produce systemic biological changes across the body. Fruhbeis et al. demonstrated that exhaustive cycle and treadmill exercise leads to a significant increase in EVs immediately post exercise which returns to baseline after 90 min. These EVs show markers consistent with exosomes and other small EVs, including CD9, CD63 and CD81 [11,189]. Consistent with these results, our group showed that after one hour of exhaustive cycle exercise, there was a significant increase in the level of EVs in the circulation compared with the resting state, with the number of EVs increasing ∼2 to ∼4 fold before returning to pre-exercise levels within 4 h of recovery [10]. Markers highly enriched in EVs, including ACTN4, ADAM10, Alix, ANAX11 and CD81, were increased after exercise, again showing the release of EVs induced by exercise is largely composed of exosomes and other small EVs. These exercise-induced EVs demonstrated tropism for the liver mediated via an increase in adhesion proteins which were transferred to liver cells when treated with exercise-induced EVs from mice. A number of proteins have been observed in exercise-induced EVs, including CD36, flotillin-1, alpha-sarcoglycan, HSP72 and Aβ; however, the biological functions of these various proteins are currently unknown [192]. We characterized 322 proteins which differed between rest and exercise, including several novel myokines which are involved in a range of biological processes, including cell metabolism—specifically, several enzymes involved in the glycolytic pathway, and the transfer of metabolites, which may allow cells to share resources during the high energy demands of exercise [10].

### 6.1. AD Pathology

While the role exercise-induced EVs may play in providing benefits to patients with neurodegenerative diseases such as AD is unknown, we speculate that it may be driven by a number of pathways. These may be activated by the various protein and non-coding mRNAs contained within the EVs. One of these potential mechanisms may be mediated by heat shock protein 70 (HSP70), which was shown to be actively secreted via exosomes both in response to stress (heat stress), but also under basal conditions [199]. Heat shock proteins are a family of intracellular proteins found in all eukaryotes and prokaryotes. They have a wide range of functions, including maintenance of cellular homeostasis and promoting cell survival. HSP70 facilities the folding of proteins via an ATP-dependent mechanism, prevents aggregation of aberrantly folded proteins and aids in protein trafficking [200,201]. A number of studies have also demonstrated that HSP70 plays a role in neurodegeneration. For instance, Sun et al. showed HSP70 had a neuroprotective effect in APP/PS1 mice, rescuing deficits in the spontaneous Y maze, the novel object test and the Morris water maze. This improvement in cognition was accompanied by a reduction in Aβ plaques, a finding which has been replicated in a number of other studies [202,203]. This reduction in Aβ pathology occurs through a number of mechanisms, including inhibition of Aβ aggregation, reduced formation of Aβ through APP binding and proteasomal degradation [204]. HSP70 has also been shown to reduce levels of phosphorylated tau through proteasomal degradation and de-phosphorylation [205].

### 6.2. Cognitive Function and Hippocampal Neurogenesis

Exercise-induced EVs may also deliver cargo which promotes hippocampal neurogenesis and so improves cognitive function. Irisin and CTSB are both myokines which have been shown to improve memory and induce adult neurogenesis in the hippocampus. CTSB has also been shown to be contained in EVs released during exercise [91]. As detailed in Section 3.1, CTSB promotes neurogenesis through the induction of BDNF [86], but also activates lysosomes [206] and has been shown to be neuroprotective in patients with AD by reducing levels of Aβ, though this remains controversial [207,208]. Whether EVs containing CTSB are able to cross the BBB and elicit a functional response is unknown; however, it is possible the increase in CTSB seen within the brain after exercise is from exercise-induced EVs which are able to cross the BBB.

### 6.3. Inflammation

As outlined in Section 4.4, exercise has been shown to have anti-inflammatory effects within the brain which correlate to cognitive function in mice [123]. These effects are mediated by the release of myokines, including IL-6, which goes on to regulate a number of anti-inflammatory cytokines, including IL-10 and TNF-α and IL-1β. Exercise-induced EVs have been shown to contain a number of these anti-inflammatory cytokines, including IL-10 and meteorin-like protein [209]. EVs containing meteorin-like protein, which has been shown to stimulate energy expenditure, improve glucose tolerance and increase expression of a number of anti-inflammatory cytokines [210], and IL-10 may provide benefits to the brain by directly regulating inflammation; activating microglia and astrocytes; and modulating peripheral inflammation, which has been shown to correlate with cognitive deficits in mild cognitive impairment and AD [211,212,213,214].

### 6.4. Metabolic Dysfunction, AD and Exercise

The association between metabolic dysfunction and a number of neurodegenerative diseases, including AD, vascular dementia and Parkinson’s disease is well established [215,216]. Conditions such as T2DM substantially increase the risk of AD, with T2DM pathology corresponding to levels of Aβ and inflammation within the brain [217]. This may be mediated through changes in insulin levels, as insulin plays a role in both the survival and function of neurons and glial cells, along with being neuroprotective by reducing levels of Aβ within the hippocampus [218,219]. Exercise is known to mitigate conditions which promote metabolic dysfunction such as T2DM and obesity, and it is becoming clearer that EVs play a role in mediating the systemic adaptions exercise confers to patients with these conditions [209,220]. That was demonstrated by Ying et al. in a study done in mice. EVs from adipose tissue macrophages in obese mice were transferred to lean mice who then developed glucose intolerance and insulin resistance; the effect was reversed when EVs were obtained from lean mice and transferred to obese mice. This effect was driven by changes in miRNA profile within EVs derived from obese compared to lean mice, particularly miR-155, which was highly abundant in obese-derived EVs [221]. These results are supported by a study by Mendonca et al. which showed exercise altered the miRNA profile of EVs released into circulation, in particular miR-22, which was negatively correlated with expression of GLUT4, a marker of insulin sensitivity [222]. This result suggests EVs play a significant role in the benefits derived from exercise for conditions which cause metabolic dysfunction, such as T2DM and obesity. It is also likely their role extends to the brain, potentially providing benefits for patients with neurodegenerative diseases; however, more work needs to be completed to confirm this hypothesis.

If exercise induces the release of EVs containing proteins such as HSP70; BDNF; irisin; CTSB; anti-inflammatory cytokines, such as IL-10 and meteorin-like protein; and other factors, including metabolites and miRNA, which promote neurogenesis and regulate metabolic function and these EVs can cross the BBB, the mechanism through which exercise provides benefits for neurodegenerative diseases, including AD, may be clear.

## Figures and Tables

**Table 1:** Exercise-induced extracellular vesicle cargo.

Model	Exercise Type	Protein	miRNA	Reference
Human	Low-Load Blood Flow restricted Resistance Exercise (BFRE)—5 Sets of knee extensions	↑ CD41, NCAM, Alix, CD25 ↓ Flotillin-1	↑ miR-182-5p, miR-16-5p, miR-1294, miR-451a, miR-363-3p ↓ miR-19b-3p, miR-17-5p, miR-221-3p, miR-150-5p, miR-340-5p, miR-21-5p	[187]
Human	Aerobic—cycling, 45 min at 55% of Vo${}_{2max}$, then 8–12 reps of knee extensions	↑ Alix, Clathrin	NA	[188]
Human	Aerobic—cycling, incremental until exhaustion	↑ CD9, CD63, CD81, CD41b, Alix,	NA	[189]
Human	Aerobic—cycling, 30 min at 55%, 20 min at 70%, and 10 min at 80%	↑ CD9, CD63, CD81, ADAM10, TSG101, + 322 proteins altered after exercise	NA	[10]
Human	Aerobic—cycling, 10 sets of 60 s at peak power	↑ CD63, HSP70	↑ miR-1-3p, miR-222-3p, miR-16-5p ↓ miR-134-3p, miR-107	[190]
Human	Aerobic/Eccentric—10 plyometric jumps, 5 sets of downhill running for 4 min at 10 kph	NA	↓ miR-31 after 24hrs	[191]
Human	Aerobic-treadmill, incremental until exhaustion	↑ Flotillin-1, HSP70, TSG101	NA	[11]
Human	Aerobic-treadmill, incremental until exhaustion	↑ CD81, TSG101	↑ miR-181a-5p, miR-1, miR-206, miR-133b, miR-499	[192]
Rat	Aerobic-swimming, 10 min increasing to 90 min per day for 4 weeks	No change in CD81, TSG101	↑ miR-3571, miR-1-3p, miR-342-5p, miR-122-5p, miR-196b-5p, miR-486, miR-208a-3p, miR-3591, miR-184, miR-760-3p, miR-99a-5p ↓ miR-191a-5p	[193]
Rat	Aerobic—swimming, 10 min increasing to 90 min per day for 4 weeks	NA	↑ miR-133a, miR-133b, miR-1, miR-206, miR-208a, miR-499	[194]
Rat	Aerobic—treadmill, 20 min at 60% daily for 2 weeks	↑ CD63	NA	[195]
Rat	Aerobic—treadmill, Low: 40 min, 14–16 m/min, Mod: 40 m, 20–22 m/min, High: 40 min, 24–26 m/min	↑ CD63	↑ miR-486, miR-191a-5p, miR-22-3p, miR-423-5p, miR-92a-3p, miR-143-3p, miR-10b-5p, miR-151-3p, miR-10a-5p, miR-3557-5p	[196]
Mouse	Aerobic—treadmill, 60 min at 5 m/min or 10 m/min daily for 4 weeks	↑ CD34	↑ miR-126	[197]
Mouse	Aerobic—treadmill, 7 m/min or 10 m/min for 300 m/day for 8 weeks	↑ CD81, Flottilin-1, TSG101	↑ miR-29b, miR-455	[198]

## Abbreviations

The following abbreviations are used in this manuscript:

AD	Alzheimer’s Disease
APP	Amyloid Precursor Protein
BBB	Blood Brain Barrier
BDNF	Brain-Derived Neurotrophic Factor
CNS	Central Nervous System
CSF	Cerebrospinal Fluid
CTSB	Cathepsin B
EV	Extracellular Vesicle
FNDC5	Fibronectin type III domain-containing protein-5
IGF-1	Insulin-like growth factor-1
NFTs	Neurofibrillary Tangles
PHF	Paired Helical Filament
NGF	Nerve Growth Factor
VEGF	Vascular Endothelial Growth Factor
